# Prevention Needs a Translator: Why Medical Journalism Matters

**DOI:** 10.1016/j.focus.2026.100528

**Published:** 2026-07-14

**Authors:** Radhika Malhotra

**Affiliations:** Department of Medicine, Rutgers New Jersey Medical School, Newark, New Jersey

The email buzzes at 12:15PM for a segment airing in 15 minutes. A producer is emailing the medical unit asking whether the script they have written is medically accurate. As the physician reviewing scripts, I pull up the study in question. One line in the script could easily turn a modest finding into a sweeping claim. In that moment, I felt the same responsibility as in clinic: translate evidence so that people can make a reasonable choice without being misled by hype, fear, or confusion.

Medical journalism matters, now more than ever. Very often people meet medicine first through headlines, broadcasts, podcasts, and social media—long before they go to see a physician.^1^^,^^2^ In a fast information ecosystem, the first story people hear can become the lens they bring into the examination room. If preventive care depends on people noticing risk early and acting on it, then the quality of the public story is not peripheral. It is prevention. When media and evidence-based care collide, tensions can distort what the public takes away, making prevention harder to deliver.

As an internal medicine and preventive medicine physician, I am trained to think at 2 levels. With individuals, clinical preventive medicine includes offering a vaccine, scheduling a screening, and helping with lifestyle change. With populations, prevention is also public health, including the policies, systems, and environments that decide whether those moments are possible, trusted, and fair. The patient–doctor relationship depends on evidence synthesis, guidelines, and regulations that translate population health findings into clinical best practice.

Even when the science is strong, prevention does not travel through guidelines alone. It travels through attention. Before the vaccine is received or a screening is booked, someone must notice an issue, understand it, and trust that the recommended action is reasonable. That is where public education comes in; medical journalism is one of its most practical tools.

I saw this firsthand during my rotation with a national medical news unit. I read new studies under embargo, wrote summaries in plain language, and reviewed medical scripts for broadcast. Although that sounds like communication work, in practice, it reflected the challenges medical experts report when working with journalists: translating nuance without losing accuracy and anticipating misinterpretation when complex evidence is compressed into a short format.^3^

This work is an extension of preventive medicine’s core mission. Preventive medicine physicians are trained in population-level thinking and translation of research into policy and practice. These are the skills that public-facing health communication demands. Creating plain-language, evidence-based public health messaging can be considered an essential activity for preventive medicine trainees. Programs such as the Stanford Global Health Media Fellowship demonstrate that structured training in science communication can equip physicians to engage media effectively and combat health misinformation,^4^ and emerging frameworks for engaging medical students in health communications offer practical models for building this capacity earlier in training.^5^ More preventive medicine physicians should be visible in these spaces, because their training uniquely positions them to ensure that public health messaging is accurate and actionable.

Embargoed access provides a small window to be thoughtful before the public conversation starts. It is also a reminder that most people will not read the full medical report. They should not have to, if the translation is done well. The task is to characterize the study accurately enough that someone who only hears the gist still comes away with something useful. This means building summaries around questions that preventive medicine cares about: what does the study show? What is missing? Who does this apply to—who does it not apply to? How big is the effect? And so what?^6^

The steepest learning curve was scriptwriting. In clinic, I am used to translating evidence into simple language. In the newsroom, I learned that the language I thought was simple is not simple at all. Broadcast is one pass. There is no pause button, no wait, what do you mean by that?, no chance to circle back and rephrase. That constraint raises the accuracy bar. A sentence that is technically correct but easy to mishear can land as misinformation anyway. A little ambiguity that would get fixed in conversation becomes the audience’s final takeaway.

This is also where the public health value becomes clear. Medical news units and health desks do classic public health jobs in real time: they scan for signals (new studies, emerging risks), sort signal from noise, translate technical findings into usable guidance, and push it through channels people use. Translation and distribution work together—a well-translated message that reaches no one is as incomplete as a widely shared headline that distorts the evidence.^7^

Journalism is not a substitute for clinical care and has pitfalls. Tight formats can oversimplify nuance, and poorly communicated uncertainty can erode trust in the messenger. The quality of medical journalism has implications for health equity. Communities with less access to trusted clinicians often rely more heavily on media for health information. Inaccurate or inaccessible health journalism can therefore widen existing knowledge gaps among historically marginalized populations.^8^^,^^9^ Conversely, well-designed, culturally responsive health communication can narrow disparities. The answer is not to step back but to build capacity on both sides of the interface.^3^ Emerging models train physicians to communicate clearly with the public and engage misinformation directly, including structured fellowships and medical education efforts focused on science communication and trust.^4^^,^^10^ These are arguments for better medical journalism, not less of it: dedicated health desks, transparent sourcing, clear explanation of limits, and rigorous medical review.

If we want prevention to work, we should treat medical journalism as part of the prevention infrastructure. That means investing in the translation layer: training clinicians to communicate evidence and uncertainty plainly, building pathways for rapid expert input when major health stories break, and supporting collaborative interventions shown to improve research coverage.^11^ When translation is done well, accurately, clearly, and appropriately humble about uncertainty, it does not just inform. It supports the everyday decisions that preventive medicine and public health depend on: someone gets vaccinated, follows through on screening, or brings the right question to their next visit. That is public health in action.

## CRediT authorship contribution statement

**Radhika Malhotra:** Conceptualization, Writing – original draft.
